# Deploying Microbial Synthesis for Halogenating and Diversifying Medicinal Alkaloid Scaffolds

**DOI:** 10.3389/fbioe.2020.594126

**Published:** 2020-10-23

**Authors:** Samuel A. Bradley, Jie Zhang, Michael K. Jensen

**Affiliations:** Novo Nordisk Foundation Center for Biosustainability, Technical University of Denmark, Lyngby, Denmark

**Keywords:** alkaloids, yeast, halogenation, plants, new-to-nature

## Abstract

Plants produce some of the most potent therapeutics and have been used for thousands of years to treat human diseases. Today, many medicinal natural products are still extracted from source plants at scale as their complexity precludes total synthesis from bulk chemicals. However, extraction from plants can be an unreliable and low-yielding source for human therapeutics, making the supply chain for some of these life-saving medicines expensive and unstable. There has therefore been significant interest in refactoring these plant pathways in genetically tractable microbes, which grow more reliably and where the plant pathways can be more easily engineered to improve the titer, rate and yield of medicinal natural products. In addition, refactoring plant biosynthetic pathways in microbes also offers the possibility to explore new-to-nature chemistry more systematically, and thereby help expand the chemical space that can be probed for drugs as well as enable the study of pharmacological properties of such new-to-nature chemistry. This perspective will review the recent progress toward heterologous production of plant medicinal alkaloids in microbial systems. In particular, we focus on the refactoring of halogenated alkaloids in yeast, which has created an unprecedented opportunity for biosynthesis of previously inaccessible new-to-nature variants of the natural alkaloid scaffolds.

## Introduction

Plants express biosynthetic pathways capable of performing a fascinating plethora of complex chemistry (Wilson and Roberts, 2014; Kutchan et al., 2015; Wurtzel and Kutchan, 2016). Consequently, many of the biologically active compounds utilized commercially, particularly pharmaceuticals, agrochemicals, flavors and fragrances, are plant-derived natural products. Pharmaceutically important classes of plant natural products include the terpenes and terpenoids (Pichersky and Raguso, 2018; Zhou and Pichersky, 2020), polyketides (Ma et al., 2009), alkaloids (Springob and Kutchan, 2009), as well as other aromatic amino acids derivatives (Springob and Kutchan, 2009). Natural products display an impressive range and density of pharmaceutical activities, many of them are FDA-approved, and more than 50% of compounds recently introduced in drug discovery pipelines are natural products or derivatives thereof (Newman and Cragg, 2016). However, most bioactive compounds possess complex structures with multiple stereocenters and oxygenated functional groups which complicate, and even preclude, total synthesis as a means of production. For this reason extraction from natural plant resources remains indispensable for sourcing bioactive compounds. For example, vincristine and vinblastine are alkaloids found in the Madagascar periwinkle (*Catharanthus roseus*) and listed by the WHO as essential medicines (World Health Organisation Model Lists of Essential Medicines, 2019). They are commercially produced by semi-synthesis, in which the biological precursors vindoline and catharanthine are extracted from *C. rosesus* and subsequently chemically coupled *in vitro* (Courdavault et al., 2020). Yet, due to the vagaries inherent to agriculture and natural habitats, the low *in planta* accumulation, and the complex mixture of chemically similar compounds found in *C. roseus*, vincristine supply for clinical usage can be unstable (Groot et al., 2018). Consequently, there is considerable interest in producing plant medicinal alkaloids, and other bioactive plant natural products, by refactoring the biosynthetic pathways in microorganisms, so-called microbial synthesis (Chang et al., 2007; Ajikumar et al., 2010; Brown et al., 2015; Fossati et al., 2015; Galanie et al., 2015; Qu et al., 2015; Li et al., 2018).

Supplying plant-derived therapeutics for human illnesses using microbial synthesis could create cheaper, greener and more reliable sources of these compounds as microbes (i) grow faster (hours for yeast as compared to months/years for plants), (ii) can be engineered to produce less complex mixtures of plant natural product, and (iii) can be cultivated using more standardized and easily scalable fermentation and downstream processing methods (Junker, 2004; Jungbauer, 2013; Buyel et al., 2015; Liu et al., 2016; Zydney, 2016; Wang et al., 2019a). Budding yeast (*Saccharomyces cerevisiae*) presents an attractive chassis for refactoring complex biosynthetic pathways of bioactive natural products, thanks to its eukaryotic cell architecture capable of supporting biosynthetic pathways that require significant endomembrane systems (e.g., P450 enzymes) or compartmentalization (Avalos et al., 2013; Zhou et al., 2016). Indeed, the seminal demonstration of artemisinic acid production in yeast (Ro et al., 2006; Paddon and Keasling, 2014) has inspired research into microbial biosynthesis of many more bioactive plant natural products. This is exemplified by the recent engineering of the native yeast mevalonate pathway to enable high flux toward geranyl pyrophosphate (GPP), and introduction of a heterologous hexanoyl-CoA biosynthetic pathway for the complete biosynthesis of cannabinoids (Luo et al., 2019). From tyrosine derivatives, biosynthesis of the common benzylisoquinoline alkaloid (BIA) precursor (S)-reticuline was a landmark achievement toward *de novo* biosynthesis of medicinal alkaloids, including hydrocodone, thebaine, stylopine, and noscapine (Galanie and Smolke, 2015; Hori et al., 2016; Li et al., 2018). Likewise, morphinan BIAs codeine and morphine have also been synthesized in yeast based on feeding substrates (R)-reticuline, salutaridine, and codeine (Fossati et al., 2015). In addition to BIAs, another major class of alkaloids are the monoterpene indole alkaloids (MIAs) derived from GPP and tryptophan (De Luca et al., 2014). Brown et al. (2015) demonstrated the *de novo* synthesis of strictosidine, the common precursor of all MIAs, in yeast through the successful refactoring of 12 heterologous enzymatic steps. Ehrenworth et al. (2015) engineered yeast to produce tetrahydrobiopterin for a mono-oxidation of tryptophan to 5-hydroxytryptophan and further onto 5-hydroxytryptamine (serotonin), which when coupled to exogenously fed secologanin enabled production of 10-hydroxystrictosidine. Further downstream of the MIA building blocks, Qu et al. (2015) demonstrated the seven-step conversion of tabersonine to the marketed anticancer precursor vindoline. In addition to BIAs and MIAs, the ergot alkaloid precursor chanoclavine-1 and the complex ergot alkaloid cycloclavine derived from tryptophan and the C5 isoprenoid unit dimethylallyl diphosphate (DMAPP) also exemplifies successful hijacking of native yeast metabolites for microbial alkaloid synthesis (Nielsen et al., 2014; Jakubczyk et al., 2015). Milne et al. (2020) recently reported the refactoring of the hallucinogenic alkaloid psilocybin biosynthetic pathway extending from an engineered shikimate pathway and coupled via tryptophan decarboxylase to yield the starting block tryptamine for four-step psilocybin biosynthesis. Beyond the refactoring of complete alkaloid biosynthetic pathways derived from natural mevalonate pathway C10 and C15 precursor units, GPP and FPP, respectively, Ignea et al. (2018) also refactored biosynthesis of 40 different C11 non-canonical terpene scaffolds, based on 2-methyl GPP production and engineered C11-specific monoterpene synthases. Lastly, tropane alkaloids derived from the arginine and polyamine metabolism biosynthesis, also should be mentioned to emphasize the versatility of yeast metabolism and cell architecture for microbial synthesis of bioactive alkaloids (Ping et al., 2019; Srinivasan and Smolke, 2019). While this review will focus on two major classes of alkaloids, BIAs and MIAs, it deserves to be mentioned that other branches of yeast’s native metabolism have been harnessed for microbial biosynthesis of non-alkaloid bioactive natural products. This includes the production of methylxanthines from the S-adenosyl methionine (SAM) *de novo* purine synthesis pathways, and adenine nucleotide pools (McKeague et al., 2016), as well as phenylpropanoids resveratrol and breviscapin produced from the shikimate pathway (Becker et al., 2003; Liu et al., 2018). Together with an elaborate review of yeast metabolism for the production of broader classes of plant natural products, this has recently been excellently covered by Chen et al. (2020).

Beyond the rational refactoring of plant natural product pathways for microbial biosynthesis of natural alkaloids, synthetic biologists are taking inspiration from medicinal chemistry campaigns investigating small-molecule drug leads, with the objective of expanding the repertoire of medicinal plant alkaloids by including their unnatural derivatives. Bioactive natural products often proceed from lead to licensing without undergoing significant modification (Ganesan, 2008). However, there is evidence that the derivatives of plant alkaloids possess new or improved pharmaceutical activities (Leggans et al., 2013; Sears and Boger, 2015; Gautam et al., 2016; Li et al., 2018). This will be of interest for (i) finding drugs against therapeutic targets that are currently considered “undruggable,” such as B-class GPCRs (Barker et al., 2013), and ii) finding improved variants against current targets—for example, 10-fluorovinblastine, 10-fluorovincristine (Sears and Boger, 2015), and halogenated noscapine variants (DeBono et al., 2015) have demonstrated improved inhibition of tumor growth relative to the natural variants. Yet, the sparsity of derivatized natural products occurs because the same complexity that bedevils total synthesis of natural products also hinders systematic chemical modification. The high number of reactive oxygenic groups makes it difficult to chemically modify specific positions without also modifying others, creating a complex product mixture. Consequently, large collections of discrete natural product derivatives are not readily available for bioactivity screening (Dandapani and Marcaurelle, 2010). It has been often hypothesized that a biological approach could generate relatively pure lead compound derivatives by harnessing the intrinsically high substrate and product specificity of enzymes. Accordingly, a number of studies have investigated the natural promiscuity of the biosynthetic pathways for medicinal plants alkaloids by feeding building block analogs or expressing building-block modifying enzymes (McCoy et al., 2006; Yerkes et al., 2008; Runguphan et al., 2010; McDonald et al., 2019). However, the slow growth rate, relatively sparse molecular toolbox, and complex regulatory systems of plants have prevented systematic engineering of the pathway as a whole, and generally limited the biological exploration of chemical space to what is achievable with the natural promiscuity. Thus, while the refactoring of these pathways into microorganisms, as described above, holds great potential to yield valuable production workhorses for existing pharmaceuticals in the near future, microbial synthesis may furthermore facilitate the engineering of natural product pathways to open up entirely novel regions of chemical space that are currently inaccessible.

**FIGURE 1:**
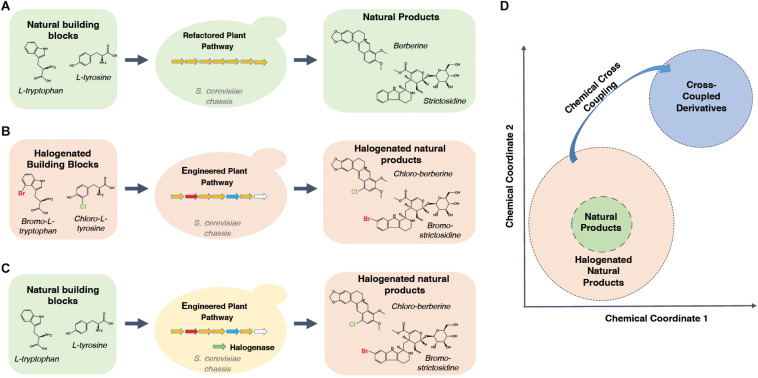
Deploying synthetic biology to access novel regions of chemical space. **(A)** Readily available bulk feedstocks can be fed to yeast strains hosting refactored plant biosynthetic pathways. Natural products can then be extracted from fermentation broth. **(B)** Engineering of the plant biosynthetic pathway within the yeast chassis can allow or improve turnover of halogenated substrate analogs. Halogenated natural product scaffolds can be extracted from the fermentation broth. Promiscuous or engineered enzyme variants in engineered plant pathways are colored by red, blue and white arrows in the middle panel. **(C)** Introduction of halogenases can yield halogenated products from natural substrates. Promiscuous or engineered enzyme variants in engineered plant pathways are colored by red, blue and white arrows in the middle panel. Halogenase is depicted by a green arrow. **(D)** Schematic depicting the halogen derivatives surrounding natural product scaffolds in a hypothetical chemical space in which more similar compounds appear closer together. Halogens can facilitate targeted cross-coupling reactions that allow “hopping” to entirely new and chemically distinct regions of chemical space.

The recent acceleration of refactoring elements of plant metabolism in microorganisms, discussed above, is creating an unprecedented resource of plant alkaloid pathways in a context more amenable their engineering. Such engineering efforts can be used to improve the pathway turnover of complex intermediate derivatives, thereby allowing researchers to produce previously inaccessible classes of compounds that can be screened for therapeutic potential. It is therefore timely to bring together these refactoring efforts and the studies reporting parts that can be integrated into future cell factories optimized for producing alkaloid derivatives. While there is a near infinite number of possible natural product derivatives, this review will first focus on the introduction of halogens (fluorine, chlorine, bromine and iodine) into natural product scaffolds, using the MIA and BIA pathways as test cases (Figures 1A–C). Secondly, although no yeast cell factories optimized for producing halogenated MIAs and BIAs yet exist, a number of *in vitro* and *in planta* studies have characterized the turnover of halogenated intermediates and developed parts relevant to achieving this goal. These will be reviewed with respect to the pathway sections that have been refactored in yeast. Finally, how such strains would represent an unprecedented opportunity to develop semi-synthetic medicinal chemistry campaigns that probe entirely new regions of chemical space will be discussed (Figure 1D).

## Halogenation in Nature and Pharmacology

**FIGURE 2:**
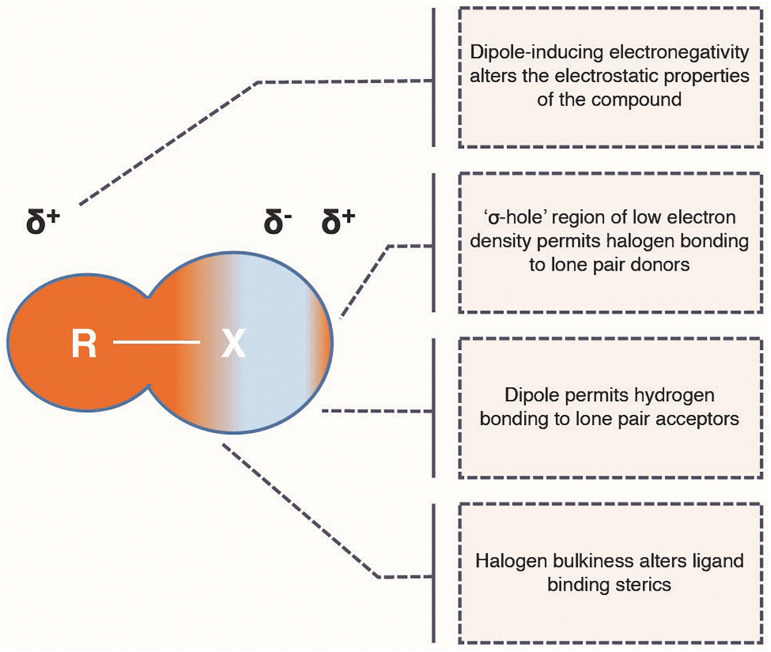
Ligand binding properties of halogen atoms. A schematic outlining the physiochemical properties of a halogen atom (X = F, Cl, Br, or I) and its potential effects on the ligand binding properties of a pharmaceutical (R = halogen-bound atom of natural product scaffold). δ^±^ denote dipole partial charges.

Halogens form group seven of the periodic table and the biologically relevant members are fluorine, chlorine, bromine and iodine. Although the prevalence of these elements in nature is becoming increasingly understood, they are not often found naturally in plant alkaloids (Runguphan et al., 2010). Conversely, halogens are highly prevalent in licensed pharmaceuticals, and often have beneficial effects on the ligand binding and their pharmacokinetic properties of human therapeutics (Fejzagić et al., 2019). This is due to a unique combination of chemical properties—bulkiness alters the sterics of ligand binding, the high electronegativity can alter the charge interactions of ligand binding, their specific orbital architectures support unique intermolecular interactions and their hydrophobicity can improve bioavailability (Figure 2). These properties and their effects on drugs have been recently reviewed (Fejzagić et al., 2019). As a consequence of these properties, organohalogens make up roughly 25% of licensed drugs (Xu et al., 2014) and 40% of all new drugs being tested (Fejzagić et al., 2019). Of these, 57% contain fluorine, 38% contain chlorine, whereas bromine and iodine make up just 5% between them (Xu et al., 2014; Fejzagić et al., 2019). In addition to directly altering pharmacokinetic properties of a compound, halogens can act as “chemical handles” for targeting further drug derivatization (Runguphan and O’Connor, 2013; Frese et al., 2016; Corr et al., 2017). They provide effective leaving groups that can allow synthetic chemists to more selectively alter the activated carbon without creating non-specific alterations at other points of the structure. This is significant when attempting to further modify complex natural products for drug use (Figure 1D) because their structural complexity makes specific substitution difficult to achieve, resulting in complex mixtures that can be costly to separate (Frese and Sewald, 2015; Schnepel and Sewald, 2017). Furthermore, FDA approval of novel compounds for therapeutic use is conditional on a pure compound being obtainable. Therefore, achieving regio-specific introduction of halogens into natural product scaffolds may also provide the key to building the natural product variant libraries that would allow the relatively unexplored derivative space surrounding natural products to be systematically probed for novel pharmaceutical activities.

However, despite considerable interest and a number of engineering studies targeting individual enzymes (Chen et al., 2006; McCoy et al., 2006; Yerkes et al., 2008; Glenn et al., 2011; Wang et al., 2019b), there has yet to be a systematic study aimed at optimizing pathway production of new-to-nature analogs of plant medicinal alkaloids. The complex chemistries of natural products that make halogenation so useful for targeting cross-coupling reactions (Figure 1D) also makes it extremely difficult to selectively halogenate the desired site in the first place (Chung and Vanderwal, 2016). Where it is possible chemically, this requires expensive catalysts (palladium) and elemental halogen, which is both toxic and energetically expensive to produce (Schnepel and Sewald, 2017; Fejzagić et al., 2019). The intrinsically high selectivity of enzymes therefore makes enzymatic halogenation a tempting alternative for achieving greener, cheaper, regio- and stereo-selective halogenation of natural products.

The potential for enzymatic halogenation emerged with the discovery of widespread halogenation in nature, which pointed toward a rich trove of halogenating enzymes operating under benign conditions (Gribble, 2003, 2018). Today, more than 6,000 naturally occurring organohalogens have been identified in a range of organisms across all three kingdoms of life, with functions including pheromones, hormones, antimicrobials, halogen recyclers, and structural proteins (Gribble, 2018). For the known organohalogens, chlorination, and bromination are the most common modifications although examples of iodination and fluorination are also reported (Gribble, 2003, 2018). Marine species and soil bacteria have provided the richest source of halogenated compounds due to the relative abundance of halogens in these habitats, particularly bromine in the ocean. A concerted effort to identify the enzymes responsible for incorporating inorganic halogen into metabolism has yielded a set of structurally and mechanistically diverse halogenases capable of catalyzing the formation of F-C, Cl-C, Br-C, and I-C bond formations (Schnepel and Sewald, 2017; Fejzagić et al., 2019).

Of the numerous halogenases, the flavin-dependent halogenases (FDHs) have been a focus for biotechnologists, due to their high substrate- and regio-specificity, and their ability to function independently of carrier proteins that bind the substrate. A subgroup of FDHs targeting the indole moiety of tryptophan are the most well studied group of FDHs, due to the prevalence of indole as a building block in both biology and synthetic chemistry (Frese and Sewald, 2015; Dhuguru and Skouta, 2020). Mechanistically, these tryptophan halogenases are similar to haem-dependent haloperoxidases and vanadium-dependent haloperoxidases in that they form a hypohalous acid (HOX) intermediate. However, unlike these other halogenases, the tryptophan FDHs do not release the HOX intermediate into solution. Instead, the HOX molecule moves through a 10 Å internal tunnel to the active site, where the halide is transferred, via a conserved lysine (Yeh et al., 2007; Flecks et al., 2008), to a specific carbon of the indole moiety of tryptophan (Karabencheva-Christova et al., 2017). Thus, this mechanism avoids the spurious halogenation associated with free HOX and achieves specific halogenation in a single step, compared to the 4–5 steps required for synthetic production of halogenated tryptophan. Indole moiety-targeting FDHs halogenating positions 5, 6 and 7 of the indole ring have been identified (Dong et al., 2005; Yeh et al., 2005; Zehner et al., 2005; Seibold et al., 2006; Fujimori et al., 2007; Heemstra and Walsh, 2008; Foulston and Bibb, 2010; Zeng and Zhan, 2011; Chang and Brady, 2013; Menon et al., 2016; Neubauer et al., 2018; Domergue et al., 2019; Ismail et al., 2019; Luhavaya et al., 2019; Lee et al., 2020; Lingkon and Bellizzi, 2020; Table 1) and, with the exception of a recently discovered brominase (Ismail et al., 2019), preferentially catalyze chlorination over bromination. Chlorination at position 4 has been observed but the responsible enzyme has not been identified (Payne et al., 2015). There are currently no known halogenases that will specifically fluorinate or iodinate tryptophan. Other common alkaloid building blocks also lack specific halogenases. However, more flavin-dependent halogenases continue to be discovered and characterized (Lingkon and Bellizzi, 2020).

**TABLE 1:** Tryptophan and indole-targeting halogenases.

Name	GenBank accession number	Organism	Reported activities	References
**Tryptophan and indole targeting halogenases**				
PrnA	AAB97504.1	*Pseudomonas fluorescens BL915*	Trp-7 halogenase	Keller et al., 2000; Dong et al., 2005
RebH	CAC93722.1	*Lechevalieria aerocolonigenes ATCC 39243*	Trp-7 halogenase	Yeh et al., 2005
KtzQ	ABV56597.1	*Kutzneria sp. 744*	Trp-7 halogenase	Fujimori et al., 2007; Heemstra and Walsh, 2008
KtzR	ABV56598.1	*Kutzneria sp. 744*	Trp-6 halogenase	Fujimori et al., 2007; Heemstra and Walsh, 2008
ThaL	ABK79936.1	*Streptomyces albogriseolus*	Trp-6 halogenase	Seibold et al., 2006
SttH	ADW94630.1	*Streptomyces toxytricini*	Trp-6 halogenase	Zeng and Zhan, 2011
BorH	AGI62217.1	Uncultured bacterium	Trp-6 halogenase	Chang and Brady, 2013; Lingkon and Bellizzi, 2020
ThdH	AGF50179.1	*Streptomyces albogriseolus MJ286−76F7*	Trp-6 halogenase	Milbredt et al., 2014
ThHal	OEJ97865.1	*Streptomyces violaceusniger SPC6*	Trp-6 halogenase	Menon et al., 2016
Tar14	WP_081761942.1	*Saccharomonospora* sp. *CNQ490*	Trp-6 halogenase	Luhavaya et al., 2019
SatH	WP_078654696.1	*Streptomyces albus*	Trp-6 halogenase	Lee et al., 2020
PyrH	AAU95674.1	*Streptomyces rugosporus NRRL 21084*	Trp-5 halogenase	Zehner et al., 2005
*Xszen*FHal	WP_038240559.1	*Xenorhabdus szentirmaii*	Trp-5 and indole halogenase	Domergue et al., 2019
BrvH	EDX81295.1	*Brevundimonas* sp. *BAL3*	Indole halogenase	Neubauer et al., 2018
*Xcc4156*	6Y1W_A	*Xanthomonas campestris* pv. campestris B100	Indole halogenase	Ismail et al., 2019

*A tabulated summary of the available FDHs targeting free tryptophan and/or indole.*

Overall, there is a convincing body of evidence to suggest that halogenated derivatives of natural product scaffolds could be a rich source of improved or entirely novel pharmaceuticals. Furthermore, the site-specific introduction of halogens can facilitate production of a near-infinite number of other derivatives via cross-coupling reactions (Figure 1D). While achieving the site selective halogenation required for this has been historically difficult, the integration of halogenases or derivatized feedstocks into natural product biosynthetic pathways is beginning to open these exciting regions of chemical space.

## Expanding Natural Product Chemical Space Through Synthetic Biology

The well-documented effects of halogen atoms on the ligand binding and pharmacokinetic properties of pharmaceuticals present a tantalizing prospect of new or improved pharmaceuticals being created by introducing halogens into these classes of already bioactive compounds. This field remains in its infancy, but the number of studies reporting microbial synthesis of new-to-nature variants of medicinal plant alkaloids is growing. This section will review the progress made toward microbial synthesis of halogenated and other new-to-nature MIA and BIA variants, and describe protein engineering efforts predating microbial refactoring that can be integrated into emerging microbial strains. While this review will focus on MIAs and BIAs, the work performed to assess and engineer derivative turnover of these pathways provides an effective template that should be possible to emulate with other plant medicinal alkaloids (Table 2).

**TABLE 2:** Enzymatically produced alkaloids from halogenated substrates.

Halogen	Halogenated substrate	Derivatized compound(s) detected	Chassis	References
**Monoterpene indole alkaloids**				
Fluorine	4-, 5-, 6-, 7-fluoroindole	Tryptophan	*In vitro*	Smith et al., 2014
	4-fluorotryptophan	Tryptamine	*In vitro*	McDonald et al., 2019
	5-fluorotryptamine	Serpentine, ajmalicine, yohimbine, vindolidine, vindoline, catharanthine	*C. roseus*	Mccoy and O’connor, 2006
	5-fluorotryptamine	Strictosidine	*In vitro*	Loris et al., 2007
	5-fluorotryptamine	Ajmalicine, tabersonine, serpentine, catharanthine	*C. roseus*	Runguphan et al., 2009
	6-fluorotryptamine	Serpentine, ajmalicine, yohimbine, akuammicine, vindolidine, catharanthine,	*C. roseus*	Mccoy and O’connor, 2006
	6-fluorotryptamine	Strictosidine	*In vitro*	Loris et al., 2007
	4-fluorotryptamine	Strictosidine, strictosidine aglycone, canthemine	*In vitro*	McCoy et al., 2006
	5-, 6-, 7-fluorotryptamine	Strictosidine, strictosidine aglycone	*In vitro*	McCoy et al., 2006
	10-, 11-fluorostrictosidine	Strictosidine aglycone	*In vitro*	Yerkes et al., 2008
Chlorine	4-, 5-, 6-, 7-chloroindole	Tryptophan	*In vitro*	Smith et al., 2014
	4-, 5-, 6-, 7-, (5,6)-(di)chlorotryptophan	Tryptamine	*In vitro*	McDonald et al., 2019
	5-chloro-L-tryptophan	Tryptamine, strictosidine, tabersonine, ajmalicine, catharanthine	*C. roseus*	Runguphan et al., 2010
	7-chloro-L-tryptophan	Tryptamine, strictosidine, dihydroakuamicine,	*C. roseus*	Runguphan et al., 2010
	5-chlorotryptamine	Strictosidine	*C. roseus*	Bernhardt et al., 2007
	5-chlorotryptamine	Ajmalicine, catharanthine, tabersonine, strictosidine, cathenamine, serpentine, isositsirikine	*C. roseus*	Runguphan and O’Connor, 2009
	7-chlorotryptamine	Dihydroakuammicine	*C. roseus*	Runguphan and O’Connor, 2013
	6-chlorotryptophan	Tryptamine	*N. benthamiana*	Fräbel et al., 2016
	7-chlorotryptophan	Tryptamine	*N. benthamiana*	Fräbel et al., 2016
	6-chlorotryptamine	Dihydroakuamicine, akuammicine, tabersonine	*C. roseus*	Runguphan and O’Connor, 2013
Bromine	4-, 5-, 6-, 7-bromoindole	Tryptophan	*In vitro*	Smith et al., 2014
	4-, 5-, 6-, 7-bromotryptophan	Tryptamine	*In vitro*	McDonald et al., 2019
	7-bromo-L-tryptophan	Dihydroakuammicine	*C. roseus*	Runguphan et al., 2010
	5-bromotryptamine	Strictosidine, ajmalicine, yohimbine, akuammicine	*C. roseus*	Bernhardt et al., 2007
	5-bromotryptamine	Ajmalicine, strictosidine, serpentine, isositsirikine	*C. roseus*	Runguphan and O’Connor, 2009
	7-bromotryptamine	Dihydroakuammicine	*C. roseus*	Runguphan and O’Connor, 2013
Iodine	7-iodoindole	Tryptophan	*In vitro*	Smith et al., 2014
	5-, 7-iodotryptophan	Tryptamine	*In vitro*	McDonald et al., 2019
**Benzylisoquinoline alkaloids**				
Fluorine	2-(4-(trifluoromethoxy)phenyl)acetaldehyde, 2-(2-fluorophenyl)acetaldehyde, 2-(3-fluorophenyl)acetaldehyde, 2-(4-fluorophenyl)acetaldehyde	Norcoclaurine	*In vitro*	Ruff et al., 2012
	3-(4-trifluoromethylphenyl)-1-propylaldehyde	Norcoclaurine	*In vitro*	Nishihachijo et al., 2014
	4−fluorophenylacetaldehyde	Norcoclaurine	*In vitro*	Pesnot et al., 2012
	3-fluoro-L-tyrosine	L-DOPA, tyramine, dopamine, norcoclaurine	*In vitro*	Wang et al., 2019b
	3-fluoro-L-tyrosine	L-DOPA, dopamine, norcoclaurine, methylcoclaurine, reticuline	*S. cerevisiae*	Li et al., 2018
Chlorine	3-chloro-L-tyrosine	Dopamine	*In vitro*	Wang et al., 2019b
	3-chloro-L-tyrosine	L-DOPA, dopamine, norcoclaurine, methylcoclaurine, reticuline	*S. cerevisiae*	Li et al., 2018
Bromine	4−bromophenylacetaldehyde	Norcoclaurine	*In vitro*	Pesnot et al., 2012
	2-bromophenylacetaldehyde	Norcoclaurine	*In vitro*	Wang et al., 2019b
	*para*-bromo-*meta*-L-tyrosine	Dopamine	*In vitro*	Wang et al., 2019b
Iodine	3-iodo-L-tyrosine	Dopamine	*In vitro*	Wang et al., 2019b
	3-iodo-L-tyrosine	L-DOPA, dopamine, norcoclaurine, methylcoclaurine, reticuline	*S. cerevisiae*	Li et al., 2018

*A tabulated summary of studies reporting enzymatic synthesis, using wild type or engineered enzymes, of new-to-nature MIAs and BIAs from halogenated substrates. Information is provided on the nature of the halogenated substrate, the natural scaffold of which halogenated derivatives were detected, and chassis in which the conversions were established.*

### New-to-Nature Monoterpenoid Indole Alkaloids

**FIGURE 3:**
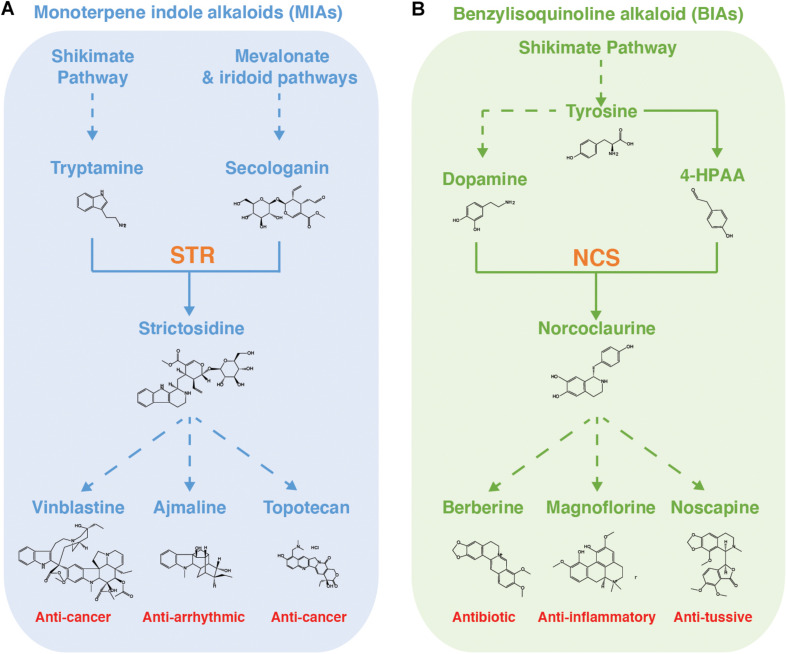
MIA and BIA biosynthetic pathways in yeast. Summaries of the **(A)** monoterpene indole alkaloid biosynthetic pathway, as refactored in yeast, and **(B)** benzylisoquinoline alkaloid biosynthetic pathway, with example compounds and, in red, their pharmaceutical applications. STR, strictosidine synthase. NCS, norcoclaurine synthase. Solid arrow denotes single reaction. Dashed arrow denotes multiple reactions.

MIA biosynthetic pathways have been popular testbeds, both *in vivo* and *in vitro*, for the biosynthesis of new-to-nature derivatives of plant medicinal alkaloids (Figure 3A). An early *in vivo* study, in which chemically synthesized tryptamines with substitutions on the indole ring were fed to *C. roseus* hairy root cultures or seedlings, observed production of fluorinated serpentine and ajmalicine, two “late-stage” MIA compounds with demonstrated pharmaceutical activities (Mccoy and O’connor, 2006). Furthermore, the authors speculated that fluorinated analogs could be widely incorporated into MIAs with minimal engineering effort due to the small size of fluorine. In another seminal study, two tryptophan halogenases (RebH and PyrH) were expressed in *C. roseus* and *de novo* production of 12-chloro-19,20-dihydroakuamicine, 10-chloroajmalicine, 15-chlorotabersonine, and 12-bromo-19,20-Dihydroakuammicine was observed (Runguphan et al., 2010). Detection of multiple “late-stage” MIA variants indicates an encouraging level of native promiscuity. However, it was observed that production of MIAs required expression of a previously identified promiscuous STR mutant (Bernhardt et al., 2007) and, interestingly, that the halogenated substrates shifted the major MIA products, most likely due to promiscuity differences forcing substrate flux into different branches (Bernhardt et al., 2007). It was further noted that accumulation of chlorotryptophan occurred, suggesting that the native tryptophan decarboxylase does not well tolerate substrate derivatives. The group were able to circumnavigate this in a follow up study in which one of the halogenases was engineered to target tryptamine instead of tryptophan (Glenn et al., 2011). This strategy may also greatly simplify separation of halogenated and unhalogenated MIAs, which would not be possible by targeting tryptophan as complete halogenation of this proteogenic amino acid would be toxic.

Other studies have more systematically investigated individual enzymes *in vitro*. The promiscuity of strictosidine synthase for various analogs of tryptophan and secologanin has been studied (McCoy et al., 2006), and it has been found that substitutions on positions 4 and 7 of the indole ring of tryptamine and smaller substitutions in general are more well tolerated (McCoy et al., 2006). Another study found that strictosidine-β-glucosidase is promiscuous for a variety of indole ring substitutions and postulated that this is because the indole moiety faces outwards from the binding site (Yerkes et al., 2008).

In parallel to these studies, a number of engineering efforts to expand substrate and product promiscuity of MIA enzymes have been undertaken. This is significant because it means that many of individual parts are already defined and ready to be combinatorially tested in microbes. Engineering to improve MIA pathway turnover of substrate analogs was first reported more than 10 years ago with the rational engineering of strictosidine synthase, through introduction of point mutations, to accept analogs of secologanin with methyl ester and vinyl positions (Chen et al., 2006). Shortly following this, a V208A point mutation in the *R. serpentina* strictosidine synthase homolog was found to improve promiscuity for tryptamines with substitutions at positions 5 and 6 of the indole ring, which the wildtype enzyme does not readily accept (Loris et al., 2007). Following this, two further *C. roseus* strictosidine synthase homolog mutants (V214M and F232L) were designed to improve promiscuity for tryptamine derivatives, and the authors subsequently observed evidence of previously unobserved brominated MIAs (Bernhardt et al., 2007). Significantly, this study also noted that the expense of purchasing chemically synthesized brominated strictosidine analogs limited the scope of the study. This expense, contrasted with the utility of chlorine and bromine in targeting site-specific cross-coupling reactions and tryptophan’s ubiquity as a synthetic building block, has driven significant interest in the more cost effective semi-synthesis or *de novo* biosynthesis of halogenated tryptophan using tryptophan halogenases (Smith et al., 2014; Frese and Sewald, 2015; Latham et al., 2018; Fejzagić et al., 2019; Table 1). Both of these approaches are viable for the more efficient production of halogenated variants of MIAs and MIA precursors. For example, a recently identified promiscuous tryptophan synthase is able to combine halogenated indole with serine to yield enantiopure samples of fluorinated, chlorinated, brominated and iodinated tryptophan (Smith et al., 2014). This enzyme could facilitate the introduction of halogens into the MIA pathway by feeding halogenated indole. Due to its lack of chirality, halogenated indole is significantly cheaper to synthesize than halogenated tryptophan and is therefore a far more attractive feedstock. This consideration of different feedstocks is particularly relevant for the introduction of fluorine into alkaloid scaffolds because only one, relatively poor, enzyme has been identified as being able to catalyze fluorination (O’Hagan and Deng, 2015). Alternatively, the introduction of bacterial tryptophan halogenases that target the indole ring of tryptophan can facilitate *de novo* production of chlorinated and brominated MIAs.

To improve the utility of promiscuous enzymes, studies have implemented rational enzyme engineering, directed evolution and synthetic biology to assess and improve substrate scope (Glenn et al., 2011; Payne et al., 2015; Shepherd et al., 2015; Neubauer et al., 2020), regio-specificity (Lang et al., 2011; Andorfer et al., 2016; Shepherd et al., 2016; Moritzer et al., 2019), stability (Payne et al., 2013; Poor et al., 2014; Minges et al., 2020) and activity (Andorfer et al., 2016, 2017; Kong et al., 2020) of FDH halogenases (Table 3). Of particular interest are a study deploying directed evolution to yield RebH variants targeting positions 5, 6, and 7 of tryptamine (Andorfer et al., 2016), which could help to avoid the tryptophan decarboxylase bottleneck, and a study in which the substrate specificity of RebH was evolved to target, albeit with lower activities, “late-stage” MIAs such as the yohimbines (Payne et al., 2015), which may allow promiscuity requirements to be eschewed entirely. These studies have relied on HPLC as a detection method, often limiting the size of the library that can be screened (Schnepel and Sewald, 2017). The recent development of novel, transcription factor-based biosensors returning a fluorescent output in response to 5- or 6-bromotryptophan (Ellefson et al., 2018) may, in combination with multiplex screening (e.g., fluorescence-activated cell sorting), underpin the screening of larger mutant libraries for optimization of the aforementioned parameters in the intracellular environment of yeast strains.

**TABLE 3:** Engineered Tryptophan FDHs.

Brief description	References
**Halogenase engineering studies**	
Tryptamine Position 7-targeting RebH mutant (Y455W).	Glenn et al., 2011
PrnA mutant (F103A) with switched activity from a position 7 to a position 5 tryptophan halogenase.	Lang et al., 2011
Improved functional RebH expression in *E. coli* through co-expression of the bacterial chaperones GroEL/GroES. Improved functional RebF expression through fusion with maltose binding protein.	Payne et al., 2013
Thermostable RebH mutant (S2P, M71V, K145M, E423D, E461G, S130L, N166S, Q494R).	Poor et al., 2014
Collection of RebH mutants that chlorinate substrates that are not accepted by the wild type enzyme (including tricyclic tryptoline, large indoles, and carbazoles).	Payne et al., 2015
RebH and PrnA mutants with expanded substrate scopes that include alternative aryl substrates.	Shepherd et al., 2015
SttH mutant (L460F/P461E/P462T) with switched activity from a position 6 to a position 5 tryptophan halogenase.	Shepherd et al., 2016
Three RebH mutants chlorinating tryptamine at positions 5 (I52H, L380F, F465C, N470S, Q494R, R509Q), 6 (I52M, S110P, S130L, N166S, L380F, S448P, Y455W, F465L, N470S, Q494R, R509Q), or 7 (N470S) with regiospecificity of at least 90%.	Andorfer et al., 2016
Bifunctional fusion enzyme consisting of a reductase (RebF) and a halogenase (RebH) showed improved yields of 7-chloro-tryptophan *in vivo*.	Andorfer et al., 2017
ThaL mutant (V52I, V82I, S360T, G469S, and S470N) with switched activity from a position 6 to a position 7 tryptophan halogenase.	Moritzer et al., 2019
Thermostable ThaL mutant (S359G, K374L, I393V) with improved activity at 25 C.	Minges et al., 2020
Bifunctional fusion enzyme consisting of a reductase (Fre) and a halogenase (XanH) showed slightly elevated halogenase activity *in vitro* compared to the two-component system.	Kong et al., 2020

*A tabulated summary of key studies reporting engineered halogenases that can be integrated into future MIA cell factories. With the exception of XanH, halogenases that do not target indole or tryptophan have been excluded.*

In addition to the ever-expanding repertoire of halogenase substrate specificities, a recent study elegantly demonstrated that these halogenases can be used in combination with a tryptophanase for biosynthesis of a halogenated indole (Fräbel et al., 2018), which is a common biosynthetic precursor. While engineering *Nicotiana benthamiana* to produce chlorinated precursors of indican dyes, the authors noted that the three tryptophan halogenases (RebH, SttH, and PyrH) did not accept indole as a substrate. They therefore allowed the halogenases to target tryptophan and expressed an *E. coli*-derived tryptophanase (TnaA) to convert this into a halogenated indole, which could then be converted, with varying efficiencies, to chloro-indican *in planta*. As indole is a common biosynthetic precursor, and a privileged heterocyclic scaffold (Dhuguru and Skouta, 2020), this system may help to unlock derivative space surrounding a number of diverse metabolites.

With the recent refactoring of the *de novo* strictosidine production in yeast (Figure 3A; Brown et al., 2015), it is expected that there will soon exist a microbial platform in which the combinatorial effects of the above-mentioned learnings can be more systematically tested. Such strains will be invaluable tools for further study because understanding of enzyme promiscuity remains patchy for the pathway leading up to strictosidine and extremely sparse for enzymes beyond this branch point. This is principally because it is only very recently that some of these modules have been fully elucidated and enzymes are still being identified (Caputi et al., 2018). The situation is further complicated by the fact that downstream substrates become increasingly complex, making derivatives difficult and expensive to chemically synthesis, leaving enzymatic synthesis as the only alternative (Figure 3A). This creates a catch-22 in which it is difficult to study *in vitro* enzyme promiscuity without the substrate analogs, yet we cannot effectively produce the substrate analogs without understanding the enzyme promiscuity. If the promiscuity of these downstream modules is to be systematically assessed and engineered *in vitro* or *in vivo*, biological platforms producing substrate analogs will have to be developed first. At first glance, source plants may look like attractive targets for these platforms. However, MIA-producing plants often possess multiple competing branches downstream of strictosidine, meaning that a small drop in turnover could cause another branch to outcompete and completely remove flux from the target branch, making it appear that there is no promiscuity. This effect was neatly demonstrated by Runguphan et al. (2010), who observed that substrate halogenation altered the major MIA products in *C. roseus*. The development of dependable, strictosidine analog-producing yeast strains, into which individual downstream MIA branches can be modularly expressed and systematically studied and engineered, will greatly accelerate our progress toward probing the derivative space surrounding MIA scaffolds.

Overall, the impressive array of pharmaceutical properties and the availability of a well-studied class of halogenases targeting direct precursors has provided the motivation and the means for more extensive new-to-nature integration of halogens into MIAs, relative to other plant medicinal alkaloids. The first production of an unnatural MIA in yeast has also already been reported—Ehrenworth et al. (2015) have reported semi-synthesis of 10-hydroxystrictosidine from yeast-derived 5-hydroxytryptamine and chemically synthesized secologanin. The principle challenge of achieving this was to engineer production of sufficient tetrahydrobiopterin cofactor for the mono-oxygenase activity yielding 5-hydroxytryptophan, which is not naturally produced in *S. cerevisiae* (Ehrenworth et al., 2015). This highlights the fact that a major challenge when microbially refactoring plant biosynthetic pathways is still often producing sufficient amounts of non-native cofactors or building blocks. A number of other significant challenges also remain, not least the enduringly low yields of microbially refactored plant biosynthetic pathways and the lack of structural information relating to enzymes downstream of strictosidine. It will therefore be some time before halogenated MIAs can be microbially produced at scale. A more attainable goal will be to produce sufficient quantities for bioactivity testing, and this would be a significant step toward unlocking the derivative space surrounding these fascinating compounds.

### New-to-Nature Benzylisoquinoline Alkaloids

BIAs are an important class of specialized plant metabolites that include the antimicrobials berberine and sanguinarine as well as the opiate analgesics (Hagel and Facchini, 2013). Several semi-synthetic opiate analogs are widely used and are found on the WHO list of essential medicines (World Health Organisation Model Lists of Essential Medicines, 2019). In higher plants, all known BIAs share a common precursor, (S)-norcoclaurine, which is the product of the first committed step in the pathway. (S)-norcoclaurine is synthesized via the norcoclaurine synthase (NCS)-catalyzed Pictet–Spengler condensation of two tyrosine derivatives (4-hydroxyphenylacetaldehyde (4-HPAA and dopamine) (Figure 3B; Facchini, 2001). As with the MIA pathway, investigations into enzymatic production of BIA derivatives began with *in vitro* investigation of individual enzyme promiscuity. NCS has been a focus of these studies due to its central position catalyzing the committed step. This enzyme has been found to accept a variety of electron rich, electron deficient and polyfunctionalized analogs of the aldehyde 4-HPAA, including fluorine-containing (Ruff et al., 2012; Nishihachijo et al., 2014) and bromine-containing (Pesnot et al., 2012) derivatives, and ketones (Lichman et al., 2017) but excluding α-substituted aldehydes. This can be rationalized through analysis of the relatively shallow active site in which the R-group is partially solvent-exposed, while the α-carbon is more deeply buried (Ilari et al., 2009; Lichman et al., 2015). However, these studies found the substrate requirements for the dopamine to be stricter, with NCS failing to turn over phenethylamines or tryptamine.

The promiscuity of early-stage BIA pathway enzymes was also recently investigated in one pot, *in vitro* enzyme cascades converting tyrosine analogs to analogs of (S)-norcoclaurine (Wang et al., 2019b). This study identified a promiscuous tyrosine decarboxylase that accepted fluorinated, chlorinated, brominated and iodinated tyrosine analogs and observed production of six non-natural BIAs, including a fluorinated analog of (S)-norcoclaurine, when this enzyme was included in the cascade. The halogenated tyrosine used in this study was purchased commercially. However, stereoselective enzymatic synthesis of fluorinated tyrosine analogs has been reported from fluoro-phenols, which are non-chiral and significantly cheaper, using tyrosine-phenol lyases (VonTersch et al., 1996; Phillips et al., 1997). Although not the endogenous route for tyrosine biosynthesis in yeast, this opens up a potential for feeding simpler precursors as a way of introducing halogens into the BIA.

More recently, *in vivo* studies have emerged in the public domain. Pyne et al. (2020) have reported construction of a novel *S. cerevisiae* strain, containing several tens of modifications principally focusing of increasing precursor supply, capable of producing 4.6 g L^–1^ of (S)-reticuline. The authors took advantage of the improved yields of this strain to search for minor products indicative of NCS promiscuity, the enzymatic gateway to probing the derivative space surrounding the tetrahydroisoquinoline (THIQ) scaffold within norcoclaurine. By screening the liquid chromatography-mass spectrometry (LC-MS) spectra of supernatants for theoretical THIQ products, the authors identified peaks consistent with THIQs formed by the condensation of dopamine and endogenous yeast carbonyl species derived from L-phenylalanine, L-tryptophan and L-leucine (Pyne et al., 2020). By cultivating strains with single amino acids as the major nitrogen source, the authors were able to increase the yield of these derivatives but also force production of a more diverse suite of aromatic- and aliphatic-derived substituted THIQs. Interestingly, a peak corresponding to the condensation of dopamine and acetaldehyde that was independent of NCS activity was also observed. This is consistent with an earlier study reporting that inorganic phosphate can catalyze aqueous formation of 1-substituted-THIQs from dopamine and aldehydes (Pesnot et al., 2011) and is reminiscent of the pH-dependent chemical coupling of secologanin and serotonin observed by Ehrenworth et al. (2015) in their hydroxystrictosidine production study. Importantly, the chemically catalyzed reactions are not enantiospecific, thus highlighting the need for robust controls when assessing product yields of microbially refactored plant pathways. Equivalent substituted THIQs derived from L-isoleucine and L-valine were not observed due to NCS’s previously reported intolerance toward α-substituted aldehydes. In a promising result for future diversification, Pesnot et al. also demonstrated that the (S)-norcoclaurine methylating enzymes, OMT and CNMT, showed activity toward all substituted THIQs.

BIA pathway engineering efforts have been concomitant with exploration of NCS promiscuity (Figure 3B). For example, screening of NCS variants with active site point mutations yielded two variants, A79I and A79F, with increased turnover of methyl-ketone and cyclohexanone 4-HPAA analogs, respectively, was recently reported by Lichman et al. (2017). This is in keeping with previous work from the same group suggesting that the active site entrance loop is a key determinant of NCS promiscuity toward the aldehyde substrate (Lichman et al., 2015). Furthermore, Li et al. (2018) reported *de novo* biosynthesis of noscapine, a BIA with anti-tussive and anticancer properties, in *S. cerevisiae*, achieving a final titer of 2.2 mg/L. The authors note that halogenated noscapine variants have shown improved bioactivity against cancer cell lines (DeBono et al., 2015; Tomar et al., 2016) and hence attempted microbial semi-synthesis noscapine derivatives by supplementing the yeast media with 16 tyrosine analogs, including 3-fluoro-tyrosine, 3-chloro-tyrosine, and 3-iodo-tyrosine (Li et al., 2018). Although no noscapine derivatives were detected, peaks matching the exact masses of 8-fluoro-reticuline, 8-chloro-reticuline and 8-iodo substituted (S)-N-methylcoclaurine were observed. The authors present (i) limited promiscuities of the native enzymes, (ii) low reaction efficiencies, and/or (iii) low substrate abundances as possible explanations for missing downstream derivatives. It has been speculated that these short-comings could be addressed by either engineering tailoring enzymes to introduce “late-stage” derivatizations, or by engineering the promiscuity of BIA enzymes such that they turn over the derivatized substrates (Tomar et al., 2016; Li et al., 2018; Srinivasan and Smolke, 2019). Drawing parallels with the tryptophan halogenases supporting halogenated MIA production, enzymatic halogenation of hydroxyisoquinoline scaffolds is also an option that warrants earnest investigation. For example, direct halogenation of the THIQ scaffold by the fungal halogenase Rdc2 (Zeng et al., 2013) or of THIQ precursors by the *Homo sapiens* thyroperoxidase (Ruf and Carayon, 2006) are both options for *de novo* production of halogenated BIAs.

## Discussion

Recent advances in the metabolic engineering toolbox have facilitated an explosion in the number of studies reporting microbial synthesis of plant medicinal alkaloids that cannot be chemically synthesized effectively. However, key challenges with both scale up of existing pathway sections and complete refactoring of both MIA and BIA extended pathways remain. Industrially competitive yeast strains able to produce even the naturally occurring plant medicinal alkaloids are still some distance into the future. Strains capable of producing industrial amounts of new-to-nature variants are yet further off. Despite this, these strains already represent an invaluable resource for the further engineering of these pathways for production of alkaloid derivatives. As described in this review, many relevant parts have already been developed, and we envision that the experimental advantages of microbial chasses will facilitate combinatorial testing of these parts to optimize derivative turnover as well as complete pathway refactoring.

Another prominent challenge will now be to progress from elegant proof-of-principles of microbial synthesis of alkaloid derivatives to more systematic explorations of the derivative space surrounding MIAs and BIAs and expanding these capabilities to include diversification of other natural product scaffolds. General approaches for achieving this will include (i) feeding of substrate analogs, (ii) pathway modification, and (iii) addition of enzymes, as recently reviewed by Cravens et al. (2019) For these non-mutually exclusive approaches to successfully produce sufficient yields for bioactivity testing of new drug leads, a number of challenges will have to be overcome. Chief among these, improving our ability to reliably engineer enzyme specificity and promiscuity would exponentially speed progress toward improved analog turnover. New computational methods designed to support protein design, such as the online webtool FuncLib (Khersonsky et al., 2018), may also aid this process. However, these approaches will also require new high-resolution structures to be published in many cases. Conversely, structure-agnostic methodologies, such as directed evolution, have already been highly successful at engineering enzyme specificities, but are limited by our ability to screen the variants (Payne et al., 2015; Andorfer et al., 2016). New biosensors targeting analogs of alkaloid precursors or intermediates may address this and help to improve analog turnover at pathway bottlenecks (Ellefson et al., 2018). Additionally, the large datasets generated by truly high throughput technologies open the door to the application of big data techniques such as machine learning, which has recently been used to successfully guide “semi-rational” protein engineering (Saito et al., 2018; Yang et al., 2019). Bioprospecting for promiscuous homologs may also prove fruitful. In the case of MIAs, the identification of two naturally occurring chlorinated alkaloids suggests that evolution may have already provided halo-tolerant enzymes and that screening alkaloid-producing plants native to halogen-rich habitats (e.g., coastal and volcanic soils) may save a significant amount of protein engineering (Al-Khdhairawi et al., 2017; Zeng et al., 2017).

Beyond improving the catalytic activity of enzymes directly catalyzing in alkaloid production, several systems level challenges will also have to be overcome. Substrate toxicity will likely be a major hurdle on the way to producing some variants in yeast. For example, it has been shown that some tryptophan analogs interfere with tryptophan synthesis in yeast and inhibit growth (Miozzari et al., 1977), and may also be erroneously incorporated into proteins. These issues could be overcome by reconsidering the halogen entry point into the pathway (Glenn et al., 2011) or engineering tryptophan tRNA synthetases to reject halogenated tryptophan. Alternatively, inspiration could be taken from a *trans*-acting aminoacyl-tRNA deacylase that selectively decouples fluoro-threonine from tRNA to prevent its integration into protein in *Streptomyces cattleya* (McMurry and Chang, 2017). Halogen salts, which are required for enzymatic halogenation, can also inhibit yeast growth. However, a number of non-conventional, halotolerant yeast strains are currently being investigated as chassis for biotechnological applications (Zaky et al., 2014; Musa et al., 2018). Although further characterization and toolbox development is still required, these strains could allow higher salt concentrations to be utilized. Functionality of the heterologously expressed plant enzymes at high salinity also remains an open question. However, we envision that both systems-level optimizations of existing chassis and further characterization of halo-tolerant yeasts could provide routes to improve the absolute yield of halogenated alkaloids, and the yield relative to the non-halogenated product, thus simplifying extraction.

Even as our understanding of how to engineer the promiscuity of these systems improves, the small number of successful studies on this topic have demonstrated the significant effort that is required to successfully produce new-to-nature variants. In addition to this, it is often difficult or expensive to source chemically synthesized intermediate analogs (Bernhardt et al., 2007) and this becomes exponentially more difficult downstream as the compounds become more complex. It will therefore be important to focus initial efforts on platform yeast strains that can produce usable quantities of multiple variants of branch point compounds, such as strictosidine in the MIA pathway and (S)-norcoclaurine in the BIA pathway. Downstream modules can then be expressed in a plug-and-play style that will allow them to be efficiently studied and engineered. Although a daunting task, several studies have reported microbial synthesis of unnatural strictosidine and (S)-norcoclaurine analogs (Chen et al., 2006; Bernhardt et al., 2007; Pesnot et al., 2012; Ehrenworth et al., 2015; Wang et al., 2019b). It should be noted that development of many of the parts required for this has already begun and these should be integrated into novel yeast strains with relative ease (Smith et al., 2014; Payne et al., 2015; Andorfer et al., 2016; McDonald et al., 2019; Moritzer et al., 2019). Thus, we argue that combinatorial effects of these optimizations are now ripe to be studied, hopefully yielding novel platform strains that produce sufficient amounts of specific derivatives to unlock new regions of chemical space.

In summary, although still relatively few, the growing number of studies reporting yeast-based microbial synthesis of plant medicinal alkaloids are providing experimentally amenable contexts in which these pathways can be further engineered. This has major implications for furthering our understanding of new-to-nature variants of natural products but will also allow the combinatorial effects of the many previous learnings from *in vitro* and *in planta* studies to be rapidly assessed. Although significant challenges still exist, if these challenges can be overcome, it is possible to envisage a new semi-synthetic approach to medicinal chemistry campaigns, specialized for diversifying natural product scaffolds. Such a system would involve microbial synthesis of medicinal alkaloid variants with halogen substitutions at specific points of the scaffold. These compounds can then be tested directly for pharmaceutical activities but also can be subjected to targeted, chemical cross-coupling reactions that can efficiently generate an almost infinite number of substituted variants. Thus, this system would combine natural mechanisms of generating diversity, i.e., using a small suite of organic building blocks combined through a large number of reactions, with synthetic mechanisms of generating diversity, i.e., using a wide range of building blocks combined with a small suite of reliable reactions (Ganesan, 2008). As these two approaches access very different regions of chemical space (Figure 1D), we foresee that this would create unprecedented opportunities for entirely new and promising regions of chemical space, possessing advantages of both the natural and synthetic spheres, to be systematically probed for pharmaceutical activities.

## Author Contributions

SB, JZ, and MJ conceived the scope of the review content and wrote the manuscript. All authors contributed to the article and approved the submitted version.

## Conflict of Interest

The authors declare that the research was conducted in the absence of any commercial or financial relationships that could be construed as a potential conflict of interest.
